# The New York Anti-Substitution Law

**Published:** 1907-08

**Authors:** 


### Abstracts and Selections.

### The New York Anti-Substitution Law.

Senator Alfred R. Page, of New York, has had enacted a bill at Albany which makes it a misdemeanor for any druggist or apothecary to dispense a different article for or in lieu of any drug or medicine specified on a physician's prescription. This bill, we are glad to announce has just been passed by the Legislature and Senate of the great State of New York and received the official signature of Governor Hughes, becoming a law to take effect on September 1, 1907.

The bill was introduced at the personal request of Mr. Charles Roome Palmele, of New York City, who, for the past five years, has been indefatigably and everlastingly hammering on this subject. The din from his persistent knocking has aroused the ire of a large number of druggists, to whom the new law says "you shall be honest in filling the physician's prescription or take the consequence." For the first offense, the punishment shall be as for a misdemeanor; for the second offense, a term of imprisonment not less than ten days nor more than one year, and a fine not less than ten dollars nor more than five hundred dollars; for the third offense, the guilty party shall be debarred from engaging in business either as proprietor, employe or agent, or otherwise in the business of apothecary, druggist or pharmacist.

This is a bill which every manufacturer of ethical proprietaries should welcome with loud acclaim, as it protects his interests, the busy physician's reputation, and the life of the sick man. All honest physicians will give Mr. Parmele the glad hand for his honest intrepidity in plunging the thorn (this bill) into the flesh of the substitutor. The prick from Parmele's thorn has made the substitutor wild-eyed; he has already lost his temper and is now snarling in despair with ebullient nihilistic spoutings, heaping malevolent anathema-maranathas upon the author of this bill, but the moon pays no attention to the nocturnal bayings of a common cur. Selah!—*Gaillard's Southern Medicine.*

Section four hundred and one of the penal code is hereby amended so as to read as follows:

Section 401. Any person who, in putting up any drug, medicine or food or preparation used in medical practice, or making up any prescription, or filling any order for drugs, medicines, food or preparation, puts any untrue label, stamp or other designation of contents upon any box, bottle or other package containing a drug, medicine, food or preparation used in medical practice, or substitutes or dispenses a different article for or in lieu of any article prescribed, ordered, or demanded, or puts up a greater or less quantity of any ingredient specified in any such prescription order or demand than that prescribed, ordered, or demanded, or otherwise deviates from the terms of the prescription, order or demand, by substituting one drug for another, is guilty of a misdemeanor; provided, however, that, except in the case of physicians' prescriptions nothing herein contained shall be deemed or construed to prevent or impair or in any manner affect the right of an apothecary, druggist, pharmacist or other person to recommend the purchase of an article other than that ordered, required or demanded, but of a similar nature, or to sell such other article in place or in lieu of an article ordered, required or demanded, with the knowledge and consent of the purchaser. Upon a second conviction for a violation of this section the offender must be sentenced to imprisonment for a term of not less than ten days nor more than one year, and to the payment of a fine of not less than ten dollars nor more than five hundred dollars. The third conviction of a violation of any of the provisions of this section, in addition to rendering the offender liable to the penalty prescribed by law for a misdemeanor, shall forfeit any right which he may possess under the law of this State at the time of such conviction to engage as proprietor, agent, employe or otherwise, in the business of an apothecary, pharmacist or druggist, or to compound, prepare or dispense prescriptions or orders for drugs, medicines or foods or preparations used in medical practice; and the offender shall be, by reason of such conviction, disqualified from engaging in any such business as proprietor, agent, employe or otherwise, or compounding, preparing or dispensing medical prescriptions or orders for drugs, medicines or foods or preparations used in medical practice.

Section 402. This act shall not affect or impair any liability, penalty or punishment under the provisions of Section 401 as the same existed prior to the time this act takes effect, but the same may be enforced, prosecuted or inflicted as fully and to the same extent as though this act had not been passed; and all actions, civil or criminal, instituted under or by virtue of said section as the same existed prior to the passage of this act, and pending immediately prior to the taking effect hereof, may be prosecuted and defended to final effect in the same manner as though this act had not been passed.

Section 403. This act shall take effect September 1, 1907.

